# The gastric mucosa of Atlantic salmon (*Salmo salar*) is abundant in highly active chitinases

**DOI:** 10.1002/2211-5463.13694

**Published:** 2023-12-04

**Authors:** Matilde Mengkrog Holen, Tina Rise Tuveng, Matthew Peter Kent, Gustav Vaaje‐Kolstad

**Affiliations:** ^1^ Center for Integrative Genetics, Department of Animal and Aquacultural Sciences, Faculty of Biosciences Norwegian University of Life Sciences Ås Norway; ^2^ Faculty of Chemistry, Biotechnology and Food Science Norwegian University of Life Sciences Ås Norway

**Keywords:** Atlantic salmon, chitin, chitinase, gastric mucosa

## Abstract

Atlantic salmon (*Salmo salar*) possesses a genome containing 10 genes encoding chitinases, yet their functional roles remain poorly understood. In other fish species, chitinases have been primarily linked to digestion, but also to other functions, as chitinase‐encoding genes are transcribed in a variety of non‐digestive organs. In this study, we investigated the properties of two chitinases belonging to the family 18 glycoside hydrolase group, namely Chia.3 and Chia.4, both isolated from the stomach mucosa. Chia.3 and Chia.4, exhibiting 95% sequence identity, proved inseparable using conventional chromatographic methods, necessitating their purification as a chitinase pair. Biochemical analysis revealed sustained chitinolytic activity against β‐chitin for up to 24 h, spanning a pH range of 2 to 6. Moreover, subsequent *in vitro* investigations established that this chitinase pair efficiently degrades diverse chitin‐containing substrates into chitobiose, highlighting the potential of Atlantic salmon to utilize novel chitin‐containing feed sources. Analysis of the gastric matrix proteome demonstrates that the chitinases are secreted and rank among the most abundant proteins in the gastric matrix. This finding correlates well with the previously observed high transcription of the corresponding chitinase genes in Atlantic salmon stomach tissue. By shedding light on the secreted chitinases in the Atlantic salmon's stomach mucosa and elucidating their functional characteristics, this study enhances our understanding of chitinase biology in this species. Moreover, the observed capacity to effectively degrade chitin‐containing materials implies the potential utilization of alternative feed sources rich in chitin, offering promising prospects for sustainable aquaculture practices.

## Graphical abstract

Atlantic salmon (*Salmo salar*) harbors 10 chitinase‐encoding genes in its genome, yet their roles remain unclear. Three of these chitinases have previously been observed transcribed in the stomach tissue, which was corroborated with proteomic analysis in the current study. Two of the stomach chitinases revealed an acidic pH optimum and high activity toward a range of chitin‐containing substrates.

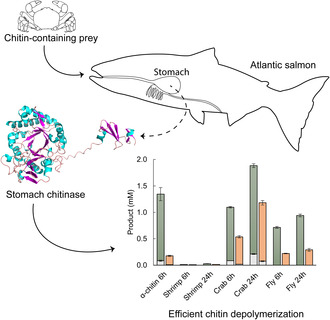

**Abbreviations**
4‐MU‐GlcNAc_2_
: 4‐Methylumbelliferyl *N*,*N*′‐diacetyl‐β‐d‐chitobioside
4‐MU‐GlcNAc_3_
: 4‐Methylumbelliferyl β‐d‐*N*,*N*′,*N*″‐triacetylchitotriose
AMCase: acidic mammalian chitinase
CAZy: carbohydrate‐active enzyme database
CBM14: carbohydrate‐binding module family 14
GalNAc: *N*‐Acetylgalactosamine
GH18: glycoside hydrolase family 18
GlcNAc: *N*‐acetylglucosamine
GlcNAc_2_
: chitobiose
HPLC: high‐performance liquid chromatography
LFQ: label‐free quantification
SmChiB: *Serratia marcescens* chitinase B
TPM: transcripts per million

Chitin is an insoluble polysaccharide, consisting of β‐1,4‐linked *N*‐acetyl‐d‐glucosamine residues. It is one of the most common biopolymers in nature and is found in three distinct allomorphic forms depending on the orientation of the chitin chains [1]: α‐chitin, β‐chitin, and γ‐chitin. In α‐chitin, the most abundant form of chitin, the polymer chains have antiparallel orientation resulting in stronger intermolecular forces compared to β‐chitin and γ‐chitin with a parallel and a mixture of parallel and antiparallel orientation of the polymer chains, respectively. Chitin functions as a structural component in algae and fungi cell walls [2, 3], in the exoskeleton of arthropods such as crustaceans and insects [4, 5], and is even hypothesized to be present in the scales and gut lining of some vertebrates, including ray‐finned fish [6, 7]. Chitin is known for its use in a large variety of applications due to its versatility as a biomaterial [8].

Chitinases are thought to play a role in at least three different processes [9], firstly in the breakdown of chitinous body structures during development, secondly they can be deployed to defend against infection of chitinous pathogens, and thirdly they can be involved in the digestion of chitin for nutrient absorption and energy production. Fish are known to express chitinases from the glycoside hydrolase 18 family (GH18), a multigene family with a conserved catalytic motif; DXXDXDXE [10], but little is known about the role of these chitinases. To our knowledge, Jeuniaux (1961) was the first to report endogenous chitinase activity in the fish gut using a β‐chitin suspension from squid pen as substrate [11]. Such gut chitinases are hypothesized to be secreted by the gastric mucosa [12, 13, 14]. These enzymes have been shown to have an acidic pH optimum, while fish that lacks an acidic stomach (e.g. zebrafish) have comparable activities at neutral pH [15]. Chitinase activity in fish intestines has not been shown to correlate with the amount of dietary chitin, but fish that swallow prey whole have shown higher chitinase activity relative to the fish that have pharyngeal teeth or other buccal cavity modifications [16]. Furthermore, fish chitinases are mainly expressed in the stomach, and stomach chitinases have different activities on various insoluble chitin substrates depending on diet [17]. This indicates that fish chitinases potentially can aid in the digestion of chitinous feed. A recent study showed that supplementation of chitin containing biomass and chitinase in the feed of Nile tilapia increased fish growth, suggesting the ability to utilize chitin degradation products as a nutrient source [18].

Wild Atlantic salmon (*Salmo salar*) is known to prey on chitinous organisms such amphipods, euphausiids, shrimp, and insects [19, 20] and possess 10 genes encoding family GH18 chitinases [21]. A better understanding of these proteins' biological functions may be valuable to the salmon industry as it searches for new, alternative feed sources. Here, we quantify the relative amount of stomach chitinases in the gastric mucosa of Atlantic salmon and isolate and characterize two of these. The results provide evidence that chitin can be degraded by these enzymes within the salmon gut.

## Results

### Sequence analysis

The Atlantic salmon genome encodes 10 chitinase‐like genes according to the NCBI RefSeq annotation (GCF_000233375.1; release 100) that all belong to family 18 of the glycoside hydrolases, as classified by the carbohydrate‐active enzyme (CAZy) database [22]. Three of these chitinase genes (hereby named *chia.3*, *chia.4*, and *chia.7*; proteins named Chia.3, Chia.4, and Chia.7, respectively) have shown stomach‐specific expression. The sequence identity of the amino acid sequences of the chitinases ranges from 60 to 95% when aligned with each other and 61 to 65% when aligned with the ortholog human acidic mammalian chitinase, AMCase (Table 1).

**Table 1:** Sequence identity (%) between Chia.3, Chia.4, and Chia.7 and AMCase (the UniProt ID is given in parenthesis) after removing the predicted signal peptide sequence (identified with signalp v.5.0 [23]).

	Chia.3 (A0A1S3L8D8)	Chia.4 (A0A1S3L8T9)	Chia.7 (A0A1S3MFN1)	AMCase (Q9BZP6)
Chia.3 (A0A1S3L8D8)	100	95	60	65
Chia.4 (A0A1S3L8T9)	95	100	60	65
Chia.7 (A0A1S3MFN1)	60	60	100	61

**Fig. 1:**
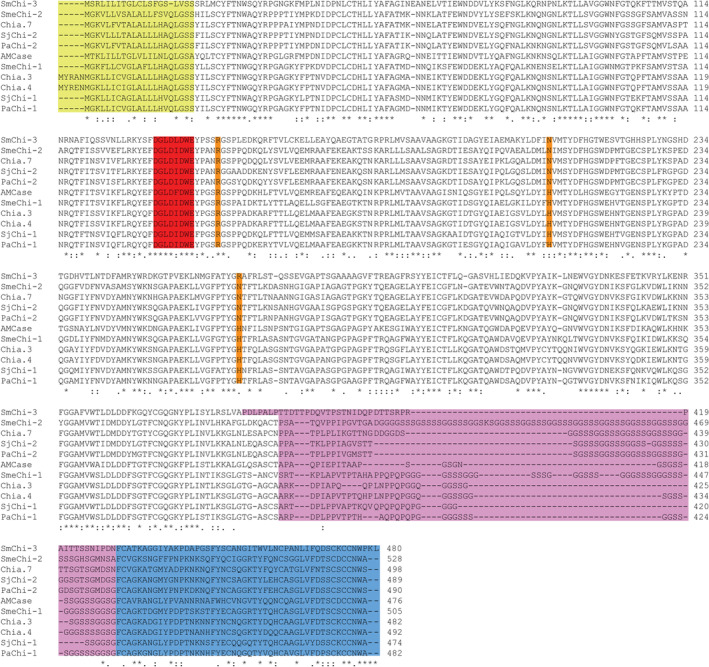
Multiple sequence alignment of stomach‐specific chitinases from Atlantic salmon (Chia.3, Chia.4, and Chia.7), Japanese Sardine (*Sardinops melanostictus*; SmeChi‐1 and SmeChi‐2), Marbled rockfish (*Sebastiscus marmoratus*; SmChi‐3), Chub mackerel (*Scomber japonicsu*; SjChi‐1 and SjChi‐2), silver croaker (*Pennahia argentata*; PaChi‐1), and human acidic mammalian chitinase (AMCase). The N‐terminal signal peptides (identified with signalp v.5.0 [23], default settings) are highlighted in yellow. The catalytic motif (DXXDXDXE) residues are highlighted in red (the glutamate represents the catalytic acid). Residues in the catalytic domain that may be important for activity in acidic environments are highlighted in orange. The C‐terminal CBM14 (identified with dbCAN2 annotation [24]) residues are highlighted in blue and the putative flexible linker, identified by inspecting the Alphafold2 predicted structures of the chitinases, are highlighted in purple.

All three chitinases share an N‐terminal signal peptide, the GH18 catalytic motif DXXDXDXE, multiple putative *N*‐Acetylgalactosamine (GalNAc) O‐glycosylation sites, and a C‐terminal domain identified as a family 14 carbohydrate‐binding module (CBM14; identified by dbCAN2 annotation [24]). A multiple sequence alignment containing the three *S. salar* stomach chitinases, several characterized acidic stomach chitinases from other fish species and the well‐characterized acidic mammalian chitinase from *H. sapiens*, revealed high similarity between the catalytic GH18 domain, but lower similarity between the CBM14 domains (Fig. 1). In addition, Chia.3, Chia.4, SmeChi‐1 from Japanese Sardine [14], SjChi‐1 from chub mackerel [13], PaChi‐1 from silver croaker [13] and AMCase, share three residues hypothesized to be important for acidic activity in the latter enzyme [25, 26]: an arginine (R) at position 145 and histidine (H) residues at positions 208 and 269 (Chia.3 amino acid numbering). Chia.7, PaChi‐2 from silver croaker [13], SmChi‐3 from marbled rockfish [27], SmeChi‐2 from Japanese Sardine [14] and PaChi‐2 from silver croaker [13] show less sequence identity to AMCase and has asparagine (N) residues at positions 208 and 269 (Chia.3 amino acid numbering). All sequences in the multiple sequence alignment are expressed in the stomach, except SmChi‐3 which is expressed in the kidneys of marbled rockfish. Interestingly, the SmChi‐3 sequence is also the most divergent of the 11 sequences analyzed, possibly indicating adaption to a different function than digestion.

### Chitinase abundance correlates with gene expression levels

**Fig. 2:**
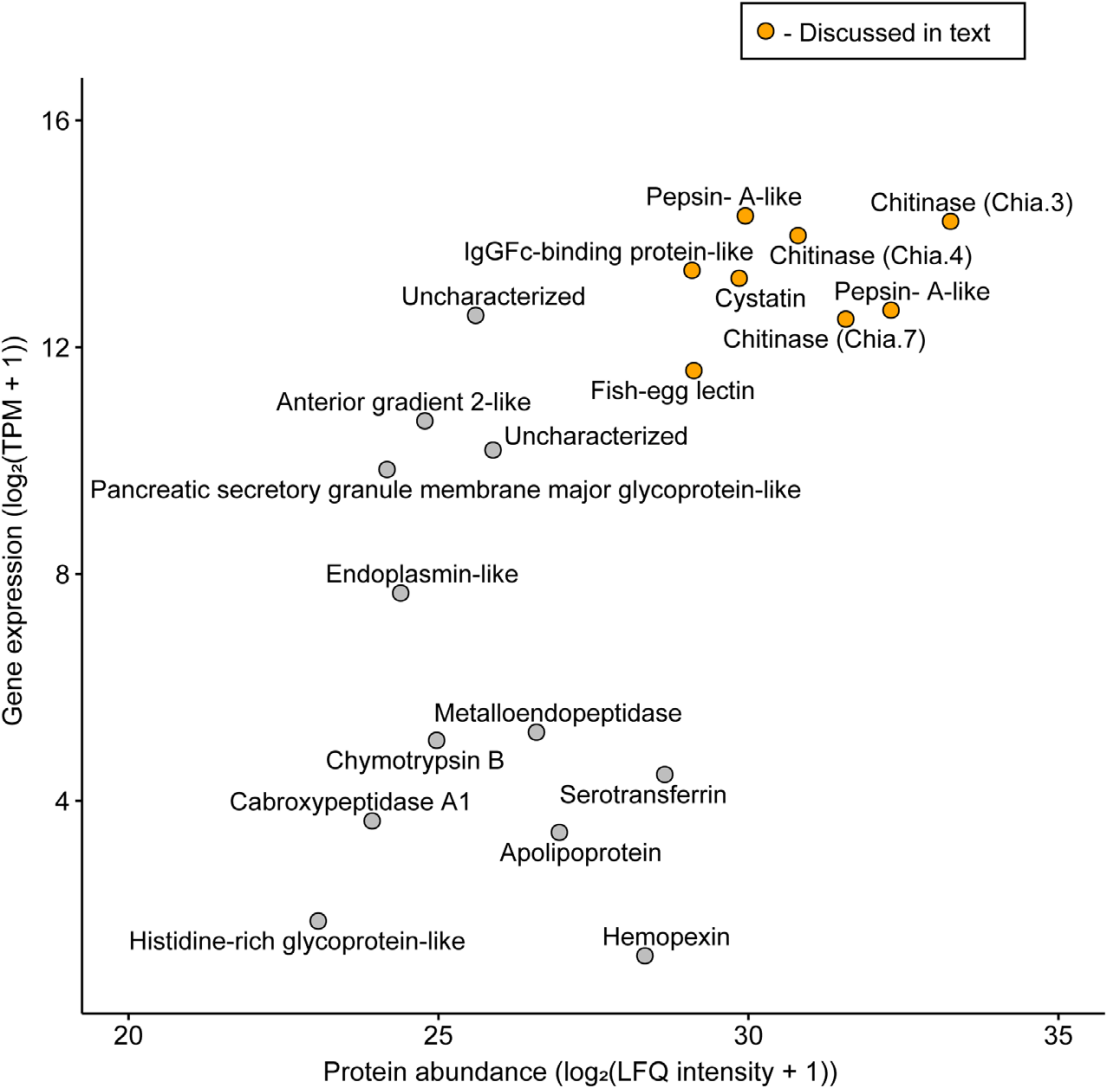
Spearman correlation between protein abundance of proteins (mean of log_2_(LFQ intensity + 1), *n* = 3) and gene expression levels (mean of log_2_(TPM + 1), *n* = 13) in Atlantic salmon stomach. The points highlighted in orange are discussed in the text. The corresponding Uniprot IDs can be found in Data S1.

Label‐free quantitative (LFQ) proteomics was used to determine the relative amount of proteins in the gastric mucosa of Atlantic salmon collected after 1 day without feed. Strikingly, Chia.3, Chia.4, and Chia.7 were among the most abundant proteins in the gastric mucosa (Fig. 2, Table 2). We took advantage of published RNA‐seq data generated from Atlantic salmon stomach tissue (ArrayExpress, E‐MTAB‐10594) to determine the correlation between the relative amount of the top 20 secreted stomach proteins and the gene expression levels of the genes coding for these proteins. The Spearman correlation (ρ) between the relative amount of proteins (log_2_(LFQ intensity + 1)) and gene expression levels (log_2_(TPM + 1), where TPM stands for transcripts per million) was 0.68 (*P*‐value = 0.0014), indicating a positive correlation between gene expression levels and protein abundance of the secreted stomach proteins (Fig. 2). Eight genes were both the most highly expressed transcripts and the most abundant proteins in the stomach including the three chitinases (Chia.3, Chia.4, and Chia.7), two Pepsin‐A‐like proteases, one IgGFc‐binding protein‐like, a cysteine protease inhibitor; cystatin, and a lectin; fish‐egg lectin. The full proteomic dataset is available as Data S1.

**Table 2:** Top 20 proteins with putative secretion encoding signal peptides in Atlantic salmon stomach mucosa, identified with label‐free quantitative proteomics.

LFQ intensity	SD	Uniprot ID	Protein name
33.26	0.84	A0A1S3L8D8	Chitinase (Chia.3)
32.30	0.55	A0A1S3NDX6	Pepsin‐A‐like
31.57	0.53	A0A1S3MFN1	Chitinase (Chia.7)
30.80	0.72	A0A1S3L8T9	Chitinase (Chia.4)
29.95	0.04	A0A1S3MIE3	Pepsin‐A‐like
29.85	1.69	B5X6A6	Cystatin
29.12	0.92	B9ENV9	Fish‐egg lectin
29.09	2.21	A0A1S3PXG0	IgGFc‐binding protein‐like
28.65	0.16	A0A1S3R1S1	Serotransferrin
28.33	0.65	A0A1S3PQV6	Hemopexin
26.95	0.24	B5XBH3	Apolipoprotein
26.58	0.34	A0A1S3KR74	Metalloendopeptidase
25.88	0.48	A0A1S3MUF1	Uncharacterized
25.60	1.07	A0A1S3LBJ3	Uncharacterized
24.97	0.12	B5XB02	Chymotrypsin B
24.78	0.31	Q2V6Q7	Anterior gradient 2‐like
24.39	0.46	A0A1S3KS25	Endoplasmin‐like
24.17	0.06	A0A1S3S2K4	Pancreatic secretory granule membrane major glycoprotein‐like
23.93	0.98	B5X8N0	Carboxypeptidase A1
23.06	0.30	A0A1S3KK24	Histidine‐rich glycoprotein‐like

### Purification and characterization of Atlantic salmon stomach chitinases

**Fig. 3:**
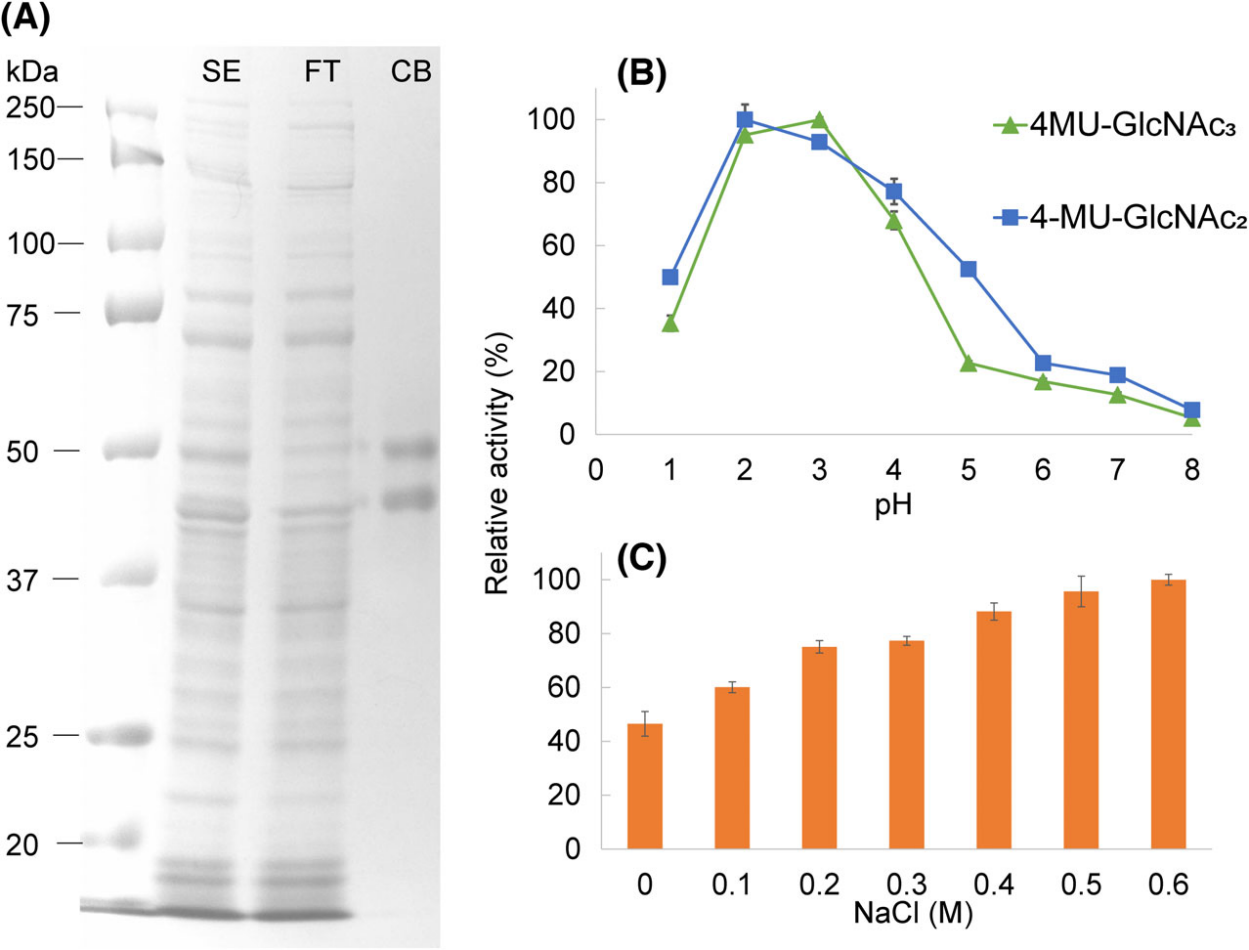
Purification and characterization of stomach chitinases from Atlantic salmon. (A) SDS/PAGE analysis of Atlantic salmon stomach chitinases purified using chitin affinity chromatography; (1) ladder (Precision Plus Protein, BioRad, Hercules, CA, USA), (2) SE: total stomach extract, (3) FT: column flow‐through, (4) CB: proteins eluted from the washed chitin affinity column. (B) Enzymatic activity at different pH values, using 100 μm 4MU‐GlcNAc_2_ (blue) and 100 μm 4MU‐GlcNAc_3_ (green) as substrate, with enzyme concentrations of 31 and 7.8 nm, respectively. The reactions were incubated for 15 min at 37 °C in 0.1 m Gly‐HCL buffer (pH 1.0–3.0) or McIlvaine's buffer (0.1 m citric acid and 0.2 m Na_2_HPO_4_, pH 4.0–8.0). Data points are joined by lines to indicate the shape of the pH activity relationship (C) Enzymatic activity with increasing NaCl concentration (0–0.6 m) using 100 μm 4MU‐GlcNAc_2_ as substrate and an enzyme concentration of 31 nm, with conditions identical as in (B), incubating the reaction in McIlvaine's buffer at pH 5. The activity was assessed in triplicate, the values shown are means ± s.d.

To capture the Atlantic salmon stomach chitinases, a stomach tissue homogenate was passed over a chitin‐affinity column and the bound proteins were eluted by pH reduction from 7 (binding buffer) to 3 (elution buffer). The eluate contained two proteins represented by two distinct bands at approximately 50 and 45 kDa (Fig. 3A). The identity of the proteins was determined by liquid chromatography with tandem mass spectrometry (LC–MS/MS), showing the presence of Chia.3 and Chia.4 in both bands. Chi.7 was also identified, but surprisingly only with a few unique peptides that barely were above the detection limit. This indicates negligible amounts of Chi.7 in the samples and that the conditions used in the affinity purification are not suited to capture this protein. All subsequent enzyme assays were performed on the chitinase eluate, mainly consisting of Chia.3 + Chia.4.

The predicted molecular weights of Chia.3 and Chia.4 after removal of the N‐terminal signal peptide are 49.6 and 50.4 kDa, respectively. The 5 kDa difference of the two bands corresponds to the molecular mass of the CBM14 domain, suggesting that the chitin binding domain has been proteolytically removed from a sub‐population of the enzymes. The proteins were not possible to separate by standard chromatographic techniques, most likely due to their highly similar protein sequence (95% alike).

To determine the influence of pH and NaCl on the activity of the chitinases, activity was determined using the chitotriose analog 4MU‐chitobioside (4MU‐GlcNAc_2_) or chitotetraose analog 4MU‐chitotrioside (4MU‐GlcNAc_3_). The highest activity was observed at pH 2 and 3 followed by a gradual decrease to pH 8 where essentially no activity could be measured (Fig. 3B). The addition of NaCl to the reaction mixture yielded a chitinase activity that increased with increasing salt concentrations, showing almost a doubling in activity from 0 to 0.6 m NaCl (Fig. 3C). The latter salt concentration approximately represents the salinity of seawater.

### Enzyme activity at acidic pH

**Fig. 4:**
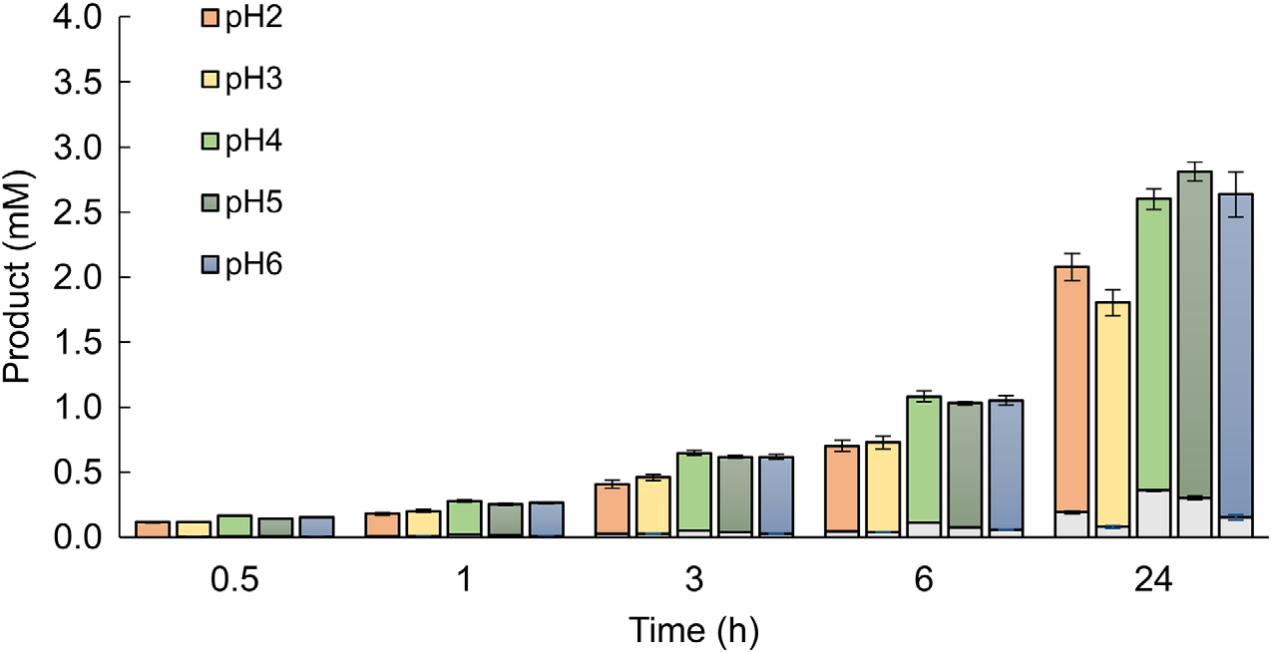
Enzyme activity at pH 2–6. Concentrations of degradations products, GlcNAc dimers (GlcNAc_2_, colored boxes) and monomers (GlcNAc, gray boxes), produced by Chia.3 + 4 when incubated with β‐chitin in pH 2–6 over 24 h are shown in a bar chart representation. The amount of degradation products was analyzed after 0.5, 1, 3, 6, and 24 h of incubation at 14 °C. The reactions were performed with an enzyme concentration of 0.2 μm and a substrate concentration of 10 mg·mL^−1^. All reactions were run in triplicates, values are means ± s.d.

Information about the pH optimum of an enzyme is sometimes not predictive of the enzyme performance over time. To determine the hydrolytic potential of the chitinase pair in relevant pH conditions using a relevant substrate, the progress of a chitin degradation reaction was followed for 24 h in reactions having pH ranging from 2 to 6 (Fig. 4). Interestingly, the highest yield after 24 h was obtained at pH 4–6, whereas highest activity was obtained at pH 2 and 3 when using the trimer and tetramer analogues as substrate (Fig. 3, panel B). The major product arising from the reactions was chitobiose (GlcNAc_2_), but some *N*‐acetylglucosamine (GlcNAc) was also observed (< 10% of the total products).

### The activity of Atlantic salmon stomach chitinases and ChiB from *Serratia marcescens* on α‐chitin‐containing organisms

The ability of the chitinase pair to depolymerize insoluble β‐chitin prompted us to investigate whether the enzymes were able to break down α‐chitin‐containing organisms commonly found in the diet of Atlantic salmon using the shell from shrimp and crab, and skin from black soldier fly pupae. To put the activity of the chitinase pair in a metabolic context, the well‐characterized chitinase from the soil bacterium *Serratia marcescens*, *Sm*ChiB was included for comparison. The experiment was done at 14 °C and pH 4.8 which are conditions similar to the Atlantic salmon stomach environment [28].

**Fig. 5:**
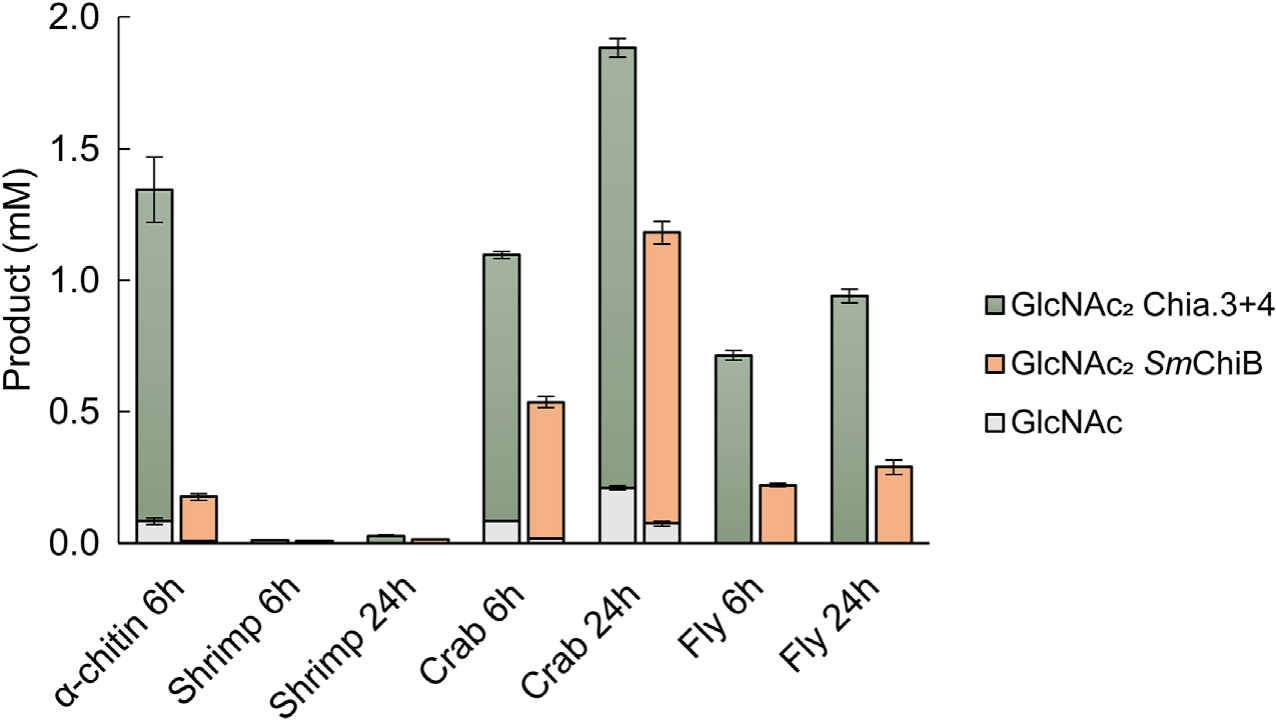
Degradation of natural α‐chitin substrates. The bar chart displays the amount of degradation products, GlcNAc dimers (GlcNAc_2_, colored boxes) and monomers (GlcNAc, gray boxes), produced by Atlantic salmon stomach chitinases (Chia.3 and Chia.4) compared to ChiB from *Serratia marcescens* (*Sm*ChiB) when incubated with α‐chitin for 6 h and shrimp shell (shrimp), crab shell (crab) and black soldier fly pupae skin (fly) for 6 and 24 h in 0.1 m sodium acetate buffer (pH 4.8) at 14 °C. The reactions were performed with an enzyme concentration of 0.5 μm and a substrate concentration of 10 mg·mL^−1^ for all substrates except black soldier fly pupae skin with a substrate concentration of 25 mg·mL^−1^. All reactions were run in triplicates, values are means ± s.d.

The results show that the chitinase pair and *Sm*ChiB were all able to partly depolymerize the chitin‐containing substrates tested (Fig. 5). The Atlantic salmon chitinase pair showed substantial activity to all substrates investigated except the dried shrimp shell and showed higher chitinolytic activity on all substrates compared to *Sm*ChiB under Atlantic salmon gastric‐like conditions, i.e. not optimal conditions for *Sm*ChiB [29]. Interestingly, *Sm*ChiB was less active on purified α‐chitin than on crab shell, while the opposite was observed for the chitinase pair after 6 h of incubation.

## Discussion

Atlantic salmon possess multiple genes encoding chitinases including three variants that are abundant as both gene transcripts and proteins in the stomach. The active proteins are secreted into the gastric mucosa but their functional role there is unknown. The stomach of Atlantic salmon works both as a “feed‐grinder” and as a first‐line defense against water‐ and feed‐borne pathogens. In theory, chitinases could play a role in the digestion of chitin‐containing feed and/or chitin‐containing pathogens such as fungi. The results show that the chitinases are present together with high levels of IgGFc‐binding‐like protein, cystatin, and fish‐egg lectin (Fig. 2), three proteins shown to play a potential role in the teleost immune system against pathogens [30, 31, 32, 33, 34]. Moreover, the Atlantic salmon stomach chitinases share many features with human AMCase [35]; they share the same conserved domains, they are abundant in the gastrointestinal tract, they are acid‐stable, and they are able to degrade crab shell chitin. AMCase has shown to play potential roles in digestion and the immune response against chitin‐containing organisms, a role that could be similar for the Atlantic salmon gastric chitinases.

In this study, Chia.3 and Chia.4 were purified from Atlantic salmon stomachs using chitin affinity chromatography, but we were unable to purify Chia.7 despite its identification by proteomic analysis. Chia.3 and Chia.4 are almost identical, while Chia.7 shows less sequence identity to the other stomach chitinases and differences in chitin‐binding capacities between the different chitinases could be the reason for not being able to isolate Chia.7 in the same conditions. Another purification approach such as ion exchange combined with hydrophobic interaction chromatography could possibly enable purification of Chi.7, as such methods have successfully been applied for other chitinases that contain partially hydrophobic substrate binding domains [29].

The combined fraction of pure Chia.3 and Chia.4 showed a pH optimum of pH 2 and 3, respectively, when using fluorogenic substrates (4MU‐chitobiose and 4MU‐chitotriose). This pH optimum for the model substrates was different from the pH optimum of pH 5 determined toward β‐chitin, a more realistic substrate for the chitinases. Differences in substrate length and composition have previously been shown to affect the chitinolytic pH optimum [36], and this underscores the need to carefully consider the substrates used when characterizing proteins. Also, the non‐natural aglycon leaving group of the 4MU‐conjugated substrates and the insoluble nature of β‐chitin may contribute to the pH preference of the enzyme. Atlantic salmon has a gastric stomach with an average pH of 4.8 depending on both feed type and time since ingestion [28], which corroborates the pH optimum data for β‐chitin. Further, the pH in the contents of the most distal part of the stomach is generally higher (pH 5) than the middle part and increases to a more neutral pH (pH 8) in the pyloric, mid, and distal intestine. Our results show that the Atlantic salmon stomach chitinase pair were highly active and stable at pH 2.0–6.0 over 24 h. This is biologically relevant as it may take up to somewhere between 24 and 48 h for the fish to empty its stomach upon ingestion [37]. A pH optimum of pH 2 when using fluorogenic substrates has previously been reported for stomach chitinases isolated from humans, mice, chicken, and pigs [35, 38, 39, 40]. Moreover, the optimum pH of stomach chitinases from Silver croaker (*Pennahia argentatus*), Marbled rockfish (*Sebastiscus marmoratus*), Red sea bream (*Pagrus major*), Japanese eel (*Anguilla japonica*), and Red scorpionfish (*Scorpaena scrofa*) have been reported to be pH 4.0–5.0, 4.0–4.5, 5.5, 4.4, and 5.0, respectively, when using colloidal chitin as a substrate [17]. This is in line with our observations.

Salmon migrate from the rivers into the sea and must adapt to a change in diet and increasing salt concentrations. The NaCl concentration of seawater corresponds to an osmolality of 1200 mOsm and the stomach chyme osmolality of rainbow trout (*Oncorhynchus mykiss*) has been reported to reach a maximum of 775 mOsm 2 h after feeding [41]. This value was reported for fish living in freshwater, and it is probably higher in seawater salmonids since it is known that salmon drink more external water for osmoregulation in seawater than in freshwater and because the gut is an important osmoregulatory organ in seawater teleost [42, 43]. Our results show that the stomach chitinases of Atlantic salmon were approximately 2‐fold more active at 0.6 m NaCl, equivalent to seawater salinity, than without any NaCl. This suggests enzyme adaptation to saline environments, a characteristic also observed for enzymes from other marine organisms [44, 45, 46]. On the other hand, it may be that the increase in activity is caused by enzyme stabilization in the specific experimental conditions used. More experiments are needed to establish the mechanism of the salt‐induced activity increase.

Furthermore, our results show that the Atlantic salmon stomach chitinases degraded chitin from shrimp shells, crab shells, and black soldier fly pupae more efficiently than ChiB from *S. marcescens*. The latter bacterium utilizes chitin as an energy source and is one of the most efficient chitin degraders out of 100 tested microorganisms [47, 48]. The higher efficiency of salmon chitinases could be a result of non‐optimal conditions for *Sm*ChiB which works best at pH 5.0–6.0 with a temperature optimum of 58 °C [29], and/or a synergy effect of the two salmon chitinases working together.

Finally, it should be noted that the potential lack of the CBM14 chitin binding domain for a sub‐population of the enzymes may have had a negative influence on the *in vitro* enzyme performance on insoluble substrates like β‐chitin and chitin‐containing exoskeletons, as it is established that CBMs increase the effective concentrations of carbohydrate active enzyme on insoluble substrates, especially when the substrates are relatively dilute. On the other hand, the CBM effect is negligible when the substrate is highly concentrated [49, 50, 51]. Thus, although possibly influencing the *in vitro* data presented here, a potential lack of a CBM may have little influence in the activity of the enzymes in the digestive system where the substrates are highly concentrated.

Recent studies show that insect meal from black soldier fly has the potential to replace fish meal in the aquaculture industry [52, 53] and that chitin and chitin degradation products can act as immunostimulants [54, 55]. Altogether, our results show that using chitin‐containing organisms as novel feed sources for farmed Atlantic salmon can be of nutritional value.

## Conclusion

Our results show that some of the most dominant proteins in the stomach of Atlantic salmon are chitinases that are capable of effectively degrading chitin or chitin‐containing substrates from various sources. The stomach chitinases are active and stable in the gastric‐like conditions of Atlantic salmon and are therefore likely to play a role in the digestion of chitin‐containing organisms commonly found in the natural diet of salmon. The results presented here can be taken into consideration when searching for novel feed ingredients in the aquaculture industry.

## Materials and methods

### Multiple sequence alignment

The amino acid sequences of Chia.3, Chia.4, Chia.7, and AMCase were downloaded from Uniprot and the alignment was calculated using Mafft with default settings in jalview v. 2.11.14. The signal peptides were predicted with signalp v.5.0 [23] using default settings. The GalNAc O‐glycosylation sites were identified with netoglyc v.4.0 [56] using default settings and the CBM14 domain was identified with dbCAN2 annotation [24].

### Chitinous substrates

β‐chitin (extracted from squid pen, Batch 20140101, France Chitin, Orange, France) was pulverized with a bead mill (Planetary Ball Mill PM 100, Retsch, Haan, Germany) to approx. 200 μm particle size. Shrimp shell was peeled off shrimps (*Pandalus Borealis*, Polar Seafood Norway, Moss, Norway) and the filling was removed from crab shell (*Cancer pagurus*, Lofotprodukt, Leknes, Norway). Both products were bought at the local food market. Shrimp shell, crab shell, and black soldier fly pupae skin (*Hermetica illucens*, a kind gift from Fraunhofer‐Gesellschaft, Munich, Germany) were dried at 105 °C overnight before the experiments were run. Shrimp‐ and crab shells were first crushed with mortar and pestle before the shells, black soldier fly pupae skin, and α‐chitin (extracted from *Pandalus borealis*, Seagarden, Avaldsnes, Norway) were pulverized with a bead mill (Planetary Ball Mill PM 100, Retsch) to approx. 200 μm particle size.

### Enzymes

Chitinases from Atlantic salmon were isolated from stomach tissue and purified as described below. Chitinase B from *Serratia marcescens* (*Sm*ChiB) was overexpressed in *Escherichia coli* and purified as previously described [57].

### Proteomic analysis of Atlantic salmon gastric mucosa

Gastric mucosa of adult Atlantic salmon (*n* = 3, two male and one female, average fish weight 2245 g) was obtained from the process plant for fish farming laboratory at The Norwegian University of Life Sciences (NMBU). The fish was starved for 1 day before they were euthanized by a blow to the head. The stomach was dissected from the fish, the gastric mucosa was scraped off and mixed with 1 mL ice‐cold phosphate buffer (20 mm sodium phosphate buffer pH 7.0, 150 mm NaCl) with 1X protease inhibitor cocktail (complete, EDTA‐free Protease Inhibitor Cocktail, Merck, Dramstadt, Germany) by pipetting and gentle vortexing. The homogenate was centrifuged at 18 500 g for 10 min at 4 °C and the supernatant was filtered through a 40‐μm cell strainer. The total protein concentration was determined with Bradford Protein Assay (Bio‐Rad) using Bovine Serum Albumin as standard. A total amount of 2 μg protein was loaded on an SDS/PAGE gel. The proteins were allowed to enter the gel, but without full separation of the proteins in the gel. The gel was stained with Coomassie Brilliant Blue R‐250 (Bio‐Rad) and de‐stained before the region of the gel containing proteins was cut out for in‐gel digestion, essentially performed as described by Shevchenko et al. [58]. In brief, proteins were reduced with DTT and alkylated with iodoacetamide before trypsinization. ZipTip C18 pipette tips (Merck, Darmstadt, Germany) were used to purify peptides, followed by drying under vacuum. The peptides were dissolved in 10 μL of 2% (v/v) acetonitrile, 0.1% (v/v) trifluoroacetic acid, and the peptide concentration was measured using NanoDrop One and used to normalize the amount of peptides injected for LC–MS/MS analysis. The LC–MS/MS analysis was performed as described by Tuveng et al. [59].

MS Raw files were analyzed using maxquant [60] version 1.6.17.0, and proteins were identified and quantified using the MaxLFQ algorithm [61]. Samples were searched against the proteome of *Salmo salar* downloaded from Uniprot (UP000087266), and a list of common contaminants (included in the maxquant software package). As variable modifications protein N‐terminal acetylation, oxidation of methionine, conversion of glutamine to pyroglutamic acid, and deamination of asparagine and glutamine were used, while carbamidomethylation of cysteine residues was used as a fixed modification. Trypsin was used as a digestion enzyme and two missed cleavages were allowed. The feature ‘Match between runs’ in maxquant, which enables identification transfer between samples based on accurate mass and retention time, was applied with default settings [61]. The results from maxquant were further processed using perseus (version 1.6.15.0) Proteins categorized as ‘only identified by site’, ‘reverse’ or as ‘contaminant’ were removed from the dataset. As an additional cut‐off criterium, proteins were only considered present if they were detected in at least two of three replicates. The LFQ intensities were log_2_‐transformed and averaged before analysis. The downstream analysis focused on proteins predicted to have a signal peptide using the *Salmo Salar* proteome (UniProt id: UP000087266).

### Comparison of protein abundance with gene expression levels

RNA‐sequencing data from the stomach of Atlantic salmon was downloaded from ArrayExpress under project number E‐MTAB‐10594. The bcbio‐nextgen pipeline (https://github.com/bcbio/bcbio‐nextgen) was used to trim, map, and count raw reads before aligning to the Atlantic salmon genome (ICSASG_v2) [62] using star [63]. Reads aligned to genes were counted with FeatureCounts [64] and transformed to transcripts per million (TPM) values to normalize for gene length. The TPM values were log_2_‐transformed and averaged (*n* = 13) before analysis. A subset of genes coding for the 20 most abundant proteins secreted in stomach mucosa was used to calculate the Spearman correlation between the gene expression levels (log_2_(TPM + 1)) and the relative protein abundance (log_2_(LFQ intensity + 1)). The statistical analysis was done using the “ggpubr” package in r v.4.0.3.

### Purification of chitinases from stomach tissue

Stomach tissue (*n* = 2, average 7.2 g per purification, two rounds of purification) from adult Atlantic salmon (one female, one male, average weight 2230 g) was obtained from the process plant for the fish farming laboratory at NMBU. The fish was euthanized by a blow to the head and the stomach was dissected from the fish. Stomach content was removed before the tissue was cut into small pieces and stored in ice‐cold phosphate buffer (20 mm sodium phosphate buffer pH 7.0, 150 mm NaCl) with 2× protease inhibitor cocktail (complete, EDTA‐free Protease Inhibitor Cocktail, Roche). The tissue was homogenized directly after dissection.

Stomach tissue was homogenized in 5 volumes of ice‐cold phosphate buffer (20 mm sodium phosphate buffer pH 7.0, 150 mm NaCl) with 2× protease inhibitor cocktail (complete, EDTA‐free Protease Inhibitor Cocktail, Roche) using TissueRuptor II (QIAGEN, 20 s on/off). The homogenate was filtered through a 40‐μm cell strainer and centrifuged at 18 500 g for 20 min at 4 °C. NaCl was added to the supernatant to get a final concentration of 1.0 m NaCl. The supernatant was filtered through a 0.22 μm sterile filter and used for chitin affinity chromatography.

The stomach extract was purified on a 1.5 cm diameter, 10 mL column packed with chitin resin slurry (New England Biolabs, Ipswich, MA, USA). The column was pre‐equilibrated with phosphate buffer (20 mm sodium phosphate buffer pH 7.0, 1.0 m NaCl) before the stomach extract was applied to the column. After washing with phosphate buffer (20 mm sodium phosphate buffer pH 7.0, 1.0 m NaCl), the chitinases were eluted with 100 mm acetic acid. A flow rate of 1 mL·min^−1^ was used at all steps. Concentration and buffer exchange of the eluted chitinases to phosphate buffer (20 mm sodium phosphate buffer pH 7.0, 150 mm NaCl) was done using a 10 kDa centrifugal filter (Macrosep Advance Centrifugal Device, 10 kDa cutoff, Pall Corporation, New York, NY, USA). The purity of the eluted chitinases was examined with SDS/PAGE and Coomassie Brilliant Blue R‐250 staining, and the proteins in the appearing bands were further identified by LC–MS/MS at the local Proteomics Core Facility (NMBU). The protein concentration was determined with Quick Start Bradford Protein Assay (Bio‐Rad) using Bovine Serum Albumin as standard.

### Confirmation of chitinase proteins with mass spectrometry

The protein bands in the de‐stained gel were cut out using a clean scalpel blade. After trimming away unstained gel, the bands were further divided into 1–2 mm cubes and transferred to clean 0.2 mL PCR tubes. 100 μL of 50% acetonitrile (ACN), 50 mm ammonium bicarbonate (ABC) was added to each tube, which was then incubated at room temperature with shaking for 10 min. After brief centrifugation, the liquid was aspirated and replaced with 200 μL 100% ACN. The tubes were incubated at room temperature for 15 min and the liquid was removed by aspiration.

The in‐gel reduction was performed by adding 50 μL 10 mm DTT, 50 mm ABC to the dried gel pieces, and incubating for 30 min at 56 °C in a thermocycler. Alkylation was performed by replacing the solution with 50 μL of 50 mm iodoacetamide, 50 mm ABC, and incubating in the dark for 20 min at room temperature.

After having removed the alkylation solution, 200 μL of 100% ACN was added and the tubes were incubated at room temperature for 15 min, followed by liquid removal and brief air drying of the gel pieces. The tubes were put on ice, and 30 μL ice‐cold trypsin solution (13 ng·μL^−1^, in 50 mm ABC) was added to each tube. The gel pieces were allowed to swell for a total of 90 min on ice, with occasional checks to ensure that they were completely covered with the digestion solution. Finally, the tubes were transferred to a thermocycler and incubated overnight at 37 °C.

Trypsin digestion was terminated by adding 50 μL TFA solution (final concentration 0.2%), and the tubes were sonicated for 10 min in a water bath sonicator. After brief centrifugation, the liquid was transferred to a clean tube. 50 μL of 0.1% TFA was added to the gel pieces and the sonication step was repeated. After combining the two extracts, peptides were purified using STAGE spin‐tips, as previously described [65]. Eluted peptides were dried in an Eppendorf Concentrator Plus vacuum centrifuge and dissolved in loading solution (0.05% TFA, 2% ACN in Milli‐Q water) before LC–MS/MS analysis.

Samples were loaded onto a trap column (Acclaim PepMap100, C_18_, 5 μm, 100 Å, 300 μm i.d. × 5 mm, Thermo Fischer Scientific, Waltham, MA, USA) and backflushed onto a 50 cm analytical column (Acclaim PepMap RSLC C_18_, 2 μm, 100 Å, 75 μm i.d., Thermo Fischer Scientific). Starting conditions were 96% solution A [0.1% (v/v) formic acid], 4% solution B [80% (v/v) ACN, 0.1% (v/v) formic acid]. Peptides were eluted using a flow rate of 300 nL·min^−1^ using a 70 min method, with the following gradient: from 3.2 to 10% B in 3 min, 10 to 35% B in 44 min and 35 to 60% B in 3 min, followed by a 5 min wash at 80% B and a 15 min equilibration at 4% B. The Q‐Exactive mass spectrometer was operated in data‐dependent acquisition (DDA) mode using a Top10 DDA method, where acquisition alternates between orbitrap‐MS and higher‐energy collisional dissociation (HCD) orbitrap‐MS/MS acquisition of the 10 most intense precursor ions. Only charge states 2–5 were selected for fragmentation, and the normalized collision energy (NCE) was set to 28. The selected precursor ions were excluded for repeated fragmentation for 20 s. The resolution was set to *R* = 70 000 and *R* = 35 000 for MS and MS/MS, respectively. Automatic gain control values were set to 3 × 10^6^ and 5 × 10^4^ for MS and MSMS, respectively, with a maximum injection time of 100 and 128 ms. The MS Raw files were converted to mgf format using msconvert available from proteowizard. The mgf files were used in Mascot Daemon searches against the refseq database (downloaded 10.10.2018), supplemented with common contaminants. Carbamidomethyl (C) was set at fixed modification and Acetyl (N‐term), Deamidated (NQ), and Oxidation (M) were set as variable modifications.

### Chitinase assays with fluorogenic substrates

Chitinolytic activity was determined using a Chitinase Assay Kit (Fluorimetric, Merck, Darmstadt, Germany) with 4‐Methylumbelliferyl *N*,*N′*‐diacetyl‐β‐d‐chitobioside (4‐MU‐GlcNAc_2_) and 4‐Methylumbelliferyl β‐d‐*N*,*N′*,*N″*‐triacetylchitotriose (4‐MU‐GlcNAc_3_) according to the manufacturer's protocol. To determine the activity in different salt concentrations 31 nm of purified chitinase was incubated with 100 μm 4‐MU‐GlcNAc_2_ in McIlvaine's buffer (0.1 m citric acid and 0.2 m Na_2_HPO_4,_ pH 5) with different concentrations of NaCl (0–0.6 m) in a volume of 100 μL at 37 °C for 15 min. To determine pH optimum, 31 nm (for assay with 4‐MU‐GlcNAc_2_) or 7.8 nm (for assay with 4‐MU‐GlcNAc_3_) of purified chitinase was incubated with 100 μm substrate in 0.1 m Gly‐HCL buffer (pH 1.0–3.0) or McIlvaine's buffer (0.1 m citric acid and 0.2 m Na_2_HPO_4_, pH 4.0–8.0) in a volume of 100 μL at 37 °C for 15 min. The reaction was stopped by the addition of 200 μL 0.4 m sodium carbonate. The fluorescence of the released 4‐Methylumbelliferone (4‐MU) was measured using a fluorimeter with excitation at 360 nm and emission at 450 nm and a 4‐MU standard curve was used to quantify 4‐MU resulting from the hydrolytic reaction. The measured fluorescence was corrected for hydrolysis of the substrate without the addition of an enzyme (blank). Each reaction was performed in triplicate.

### pH and stability assay

Purified chitinase enzyme or phosphate buffer (blank) was mixed with β‐chitin in appropriate volumes of the following buffer solutions: 20 mm glycine‐HCL (pH 2 and 3), 20 mm sodium acetate (pH 4 and 5), 20 mm sodium phosphate (pH 6), to yield final concentrations of 0.2 μm (chitinase) and 10 mg·mL^−1^ (β‐chitin). The reaction mixtures were incubated at 14 °C in an Eppendorf thermomixer at 1000 rpm and samples were taken after 0.5, 1, 3, 6, and 24 h of incubation and filtered to remove β‐chitin particles and thereby stop the reaction (0.45 μm vacuum filter, Merck Millipore). To adjust the samples for chromatography and to inactivate enzymes, H_2_SO_4_ was added to a final concentration of 20 mm in the filtrate. All reactions were run in triplicates. The end products were analyzed by high‐performance liquid chromatography (HPLC; see below).

### Chitinase assay with α‐chitin, shrimp shells, crab shells, and black soldier fly pupae skin

Purified chitinase from Atlantic salmon (0.5 μm), purified ChiB from *Serratia marscecens* (0.5 μm) or phosphate buffer (blank) was mixed with α‐chitin (10 mg·mL^−1^), shrimp shell (10 mg·mL^−1^), crab shell (10 mg·mL^−1^), and black soldier fly pupae skin (25 mg·mL^−1^) in 0.1 m sodium acetate (pH 4.8), and incubated at 14 °C in an Eppendorf thermomixer at 1000 rpm (all concentrations noted in parenthesis indicate final concentrations in the reaction mixtures). Samples were withdrawn after 6 and 24 h of incubation, filtered by a 0.45 μm filter to remove the substrate particles from the reaction mixture and thereby stop the reaction. The enzyme activity was inactivated by addition H_2_SO_4_ to a final concentration of 20 mm in the filtrate. All reactions were run in triplicates. The end products were analyzed by HPLC (see below).

### High‐performance liquid chromatography

Concentrations of mono‐ and disaccharides of *N*‐acetylglucosamine (GlcNAc and GlcNAc_2_) were determined with HPLC as previously described [66].

## Conflict of interest

The authors declare no conflict of interest.

### Peer review

The peer review history for this article is available at https://www.webofscience.com/api/gateway/wos/peer‐review/10.1002/2211‐5463.13694.

## Author contributions

MMH, GV‐K, and MPK designed, conceptualized, and supervised the research. MMH performed the experiments and analyzed the data. TRT helped with performing the experiments and analyzing the data related to proteomic analysis and chitinase activity analysis. MMH, GV‐K, and TRT wrote the manuscript, and MPK edited the manuscript. All authors contributed to the final editing of the manuscript and approved the submitted version.

## Welfare statement

The experiment was conducted in accordance with Norwegian and European regulations related to animal research. Formal approval of the experiment by the Norwegian Animal Research Authority (NARA) was not required as the experimental conditions were following routine practices at the Centre for Fish Research at NMBU and no compromised welfare was expected.

## Supporting information


**Data S1.** Proteomic dataset of Atlantic salmon stomach tissue homogenate.

## Data Availability

The proteomics data have been deposited to the ProteomeXchange consortium (http://proteomecentral.proteomexchange.org) via the PRIDE [67] partner repository with the dataset identifier PXD030291.

## References

[1] Jang M‐K , Kong B‐G , Jeong Y‐I , Lee CH and Nah J‐W (2004) Physicochemical characterization of α‐chitin, β‐chitin, and γ‐chitin separated from natural resources. J Polym Sci A Polym Chem 42, 3423–3432.

[2] Bowman SM and Free SJ (2006) The structure and synthesis of the fungal cell wall. Bioessays 28, 799–808. 16927300 10.1002/bies.20441

[3] Pearlmutter NL and Lembi CA (1978) Localization of chitin in algal and fungal cell walls by light and electron microscopy. J Histochem Cytochem 26, 782–791. 722047 10.1177/26.10.722047

[4] Kramer KJ , Hopkins TL and Schaefer J (1995) Applications of solids NMR to the analysis of insect sclerotized structures. Insect Biochem Mol Biol 25, 1067–1080.

[5] Kurita K (2006) Chitin and chitosan: functional biopolymers from marine crustaceans. Marine Biotechnol 8, 203–226. 10.1007/s10126-005-0097-5 16532368

[6] Tang WJ , Fernandez J , Sohn JJ and Amemiya CT (2015) Chitin is endogenously produced in vertebrates. Curr Biol 25, 897–900. 25772447 10.1016/j.cub.2015.01.058 PMC4382437

[7] Nakashima K , Kimura S , Ogawa Y , Watanabe S , Soma S , Kaneko T , Yamada L , Sawada H , Tung C‐H , Lu T‐M *et al*. (2018) Chitin‐based barrier immunity and its loss predated mucus‐colonization by indigenous gut microbiota. Nat Commun 9, 3402. 30143642 10.1038/s41467-018-05884-0 PMC6109156

[8] Lv J , Lv X , Ma M , Oh D‐H , Jiang Z and Fu X (2023) Chitin and chitin‐based biomaterials: a review of advances in processing and food applications. Carbohydr Polym 299, 120142. 36876773 10.1016/j.carbpol.2022.120142

[9] Rathore AS and Gupta RD (2015) Chitinases from bacteria to human: properties, applications, and future perspectives. Enzyme Res 2015, 791907. 26664744 10.1155/2015/791907 PMC4668315

[10] Hussain M and Wilson JB (2013) New paralogues and revised time line in the expansion of the vertebrate GH18 family. J Mol Evol 76, 240–260. 23558346 10.1007/s00239-013-9553-4

[11] Jeuniaux C (1961) Chitinase: an addition to the list of hydrolases in the digestive tract of vertebrates. Nature 192, 135–136. 14451758 10.1038/192135a0

[12] Dandrifosse G and Schoffeniels E (1965) Existence of a mechanism of chitinase transport across cell membranes. Biochim Biophys Acta 94, 165–174. 14273396

[13] Kakizaki H , Ikeda M , Fukushima H and Matsumiya M (2015) Distribution of chitinolytic enzymes in the organs and cDNA cloning of chitinase isozymes from the stomach of two species of fish, chub mackerel (*Scomber japonicus*) and silver croaker (*Pennahia argentata*). Open J Mar Sci 5, 398–411.

[14] Kawashima S , Ikehata H , Tada C , Ogino T , Kakizaki H , Ikeda M , Fukushima H and Matsumiya M (2016) Stomach chitinase from Japanese sardine *Sardinops melanostictus*: purification, characterization, and molecular cloning of chitinase isozymes with a long linker. Mar Drugs 14, 22. 26805857 10.3390/md14010022 PMC4728518

[15] Teng Z , Sun C , Liu S , Wang H and Zhang S (2014) Functional characterization of chitinase‐3 reveals involvement of chitinases in early embryo immunity in zebrafish. Dev Comp Immunol 46, 489–498. 24968080 10.1016/j.dci.2014.06.008

[16] Lindsay GJH (1984) Distribution and function of digestive tract chitinolytic enzymes in fish. J Fish Biol 24, 529–536.

[17] Ikeda M , Kakizaki H and Matsumiya M (2017) Biochemistry of fish stomach chitinase. Int J Biol Macromol 104, 1672–1681. 28365290 10.1016/j.ijbiomac.2017.03.118

[18] Agbohessou PS , Mandiki SNM , Mbondo Biyong SR , Cornet V , Nguyen TM , Lambert J , Jauniaux T , Lalèyè PA and Kestemont P (2022) Intestinal histopathology and immune responses following *Escherichia coli* lipopolysaccharide challenge in Nile tilapia fed enriched black soldier fly larval (BSF) meal supplemented with chitinase. Fish Shellfish Immunol 128, 620–633. 36038101 10.1016/j.fsi.2022.08.050

[19] Jacobsen J (2001) Feeding habits of wild and escaped farmed Atlantic salmon, *Salmo salar* L., in the Northeast Atlantic. ICES J Mar Sci 58, 916–933.

[20] Rikardsen AH , Haugland M , Bjørn PA , Finstad B , Knudsen R , Dempson JB , Holst JC , Hvidsten NA and Holm M (2004) Geographical differences in marine feeding of Atlantic salmon post‐smolts in Norwegian fjords. J Fish Biol 64, 1655–1679.

[21] Holen MM , Vaaje‐Kolstad G , Kent MP and Sandve SR (2023) Gene family expansion and functional diversification of chitinase and chitin synthase genes in Atlantic salmon (*Salmo salar*). G3 13, jkad069. 36972305 10.1093/g3journal/jkad069 PMC10234404

[22] Drula E , Garron M‐L , Dogan S , Lombard V , Henrissat B and Terrapon N (2022) The carbohydrate‐active enzyme database: functions and literature. Nucleic Acids Res 50, D571–D577. 34850161 10.1093/nar/gkab1045 PMC8728194

[23] Nielsen H , Engelbrecht J , Brunak S and von Heijne G (1997) Identification of prokaryotic and eukaryotic signal peptides and prediction of their cleavage sites. Protein Eng 10, 1–6. 10.1093/protein/10.1.1 9051728

[24] Zhang H , Yohe T , Huang L , Entwistle S , Wu P , Yang Z , Busk PK , Xu Y and Yin Y (2018) dbCAN2: a meta server for automated carbohydrate‐active enzyme annotation. Nucleic Acids Res 46, W95–W101. 29771380 10.1093/nar/gky418 PMC6031026

[25] Olland AM , Strand J , Presman E , Czerwinski R , Joseph‐McCarthy D , Krykbaev R , Schlingmann G , Chopra R , Lin L , Fleming M *et al*. (2009) Triad of polar residues implicated in pH specificity of acidic mammalian chitinase. Protein Sci 18, 569–578. 19241384 10.1002/pro.63 PMC2760363

[26] Bussink AP , Vreede J , Aerts JMFG and Boot RG (2008) A single histidine residue modulates enzymatic activity in acidic mammalian chitinase. FEBS Lett 582, 931–935. 18294964 10.1016/j.febslet.2008.02.032

[27] Watanabe M , Kakizaki H , Tsukamoto T , Fujiwara M , Fukushima H , Ueda M and Matsumiya M (2018) Distribution of chitinolytic enzyme in the organs and molecular cloning of a novel chitinase gene from the kidney of marbled rockfish *Sebastiscus marmoratus* . Abbottempo 9, 36–51.

[28] Krogdahl Å , Sundby A and Holm H (2015) Characteristics of digestive processes in Atlantic salmon (*Salmo salar*). Enzyme pH optima, chyme pH, and enzyme activities. Aquaculture 449, 27–36.

[29] Brurberg MB , Nes IF and Eijsink VGH (1996) Comparative studies of chitinases A and B from *Serratia marcescens* . Microbiology 142 (Pt 7), 1581–1589. 8757722 10.1099/13500872-142-7-1581

[30] Elvitigala DAS and Lee J (2021) Identification and molecular profiling of a novel homolog of cystatin C from rock bream (*Oplegnathus fasciatus*) evidencing its transcriptional sensitivity to pathogen infections. Mol Biol Rep 48, 4933–4942. 34041676 10.1007/s11033-021-06415-9

[31] Kim B‐S , Nam B‐H , Kim J‐W , Park H‐J , Song J‐H and Park C‐I (2011) Molecular characterisation and expression analysis of a fish‐egg lectin in rock bream, and its response to bacterial or viral infection. Fish Shellfish Immunol 31, 1201–1207. 22056500 10.1016/j.fsi.2011.10.024

[32] Peatman E , Lange M , Zhao H and Beck BH (2015) Physiology and immunology of mucosal barriers in catfish (*Ictalurus* spp.). Tissue Barriers 3, e1068907. 26716071 10.1080/21688370.2015.1068907 PMC4681283

[33] Wang Y , Bu L , Yang L , Li H and Zhang S (2016) Identification and functional characterization of fish‐egg lectin in zebrafish. Fish Shellfish Immunol 52, 23–30. 26975412 10.1016/j.fsi.2016.03.016

[34] Zhang K , Liu X , Li X , Liu Y , Yu H , Liu J and Zhang Q (2020) Antibacterial functions of a novel fish‐egg lectin from spotted knifejaw (*Oplegnathus punctatus*) during host defense immune responses. Dev Comp Immunol 111, 103758. 32502504 10.1016/j.dci.2020.103758

[35] Boot RG , Blommaart EF , Swart E , Ghauharali‐van der Vlugt K , Bijl N , Moe C , Place A and Aerts JM (2001) Identification of a novel acidic mammalian chitinase distinct from chitotriosidase. J Biol Chem 276, 6770–6778. 11085997 10.1074/jbc.M009886200

[36] Barad BA , Liu L , Diaz RE , Basilio R , Van Dyken SJ , Locksley RM and Fraser JS (2020) Differences in the chitinolytic activity of mammalian chitinases on soluble and insoluble substrates. Protein Sci 29, 966–977. 31930591 10.1002/pro.3822 PMC7096708

[37] Aas TS , Sixten HJ , Hillestad M , Sveier H , Ytrestøyl T , Hatlen B and Åsgård T (2017) Measurement of gastrointestinal passage rate in Atlantic salmon (*Salmo salar*) fed dry or soaked feed. Aquac Rep 8, 49–57.

[38] Ohno M , Kimura M , Miyazaki H , Okawa K , Onuki R , Nemoto C , Tabata E , Wakita S , Kashimura A , Sakaguchi M *et al*. (2016) Acidic mammalian chitinase is a proteases‐resistant glycosidase in mouse digestive system. Sci Rep 6, 37756. 27883045 10.1038/srep37756 PMC5121897

[39] Tabata E , Kashimura A , Wakita S , Ohno M , Sakaguchi M , Sugahara Y , Kino Y , Matoska V , Bauer PO and Oyama F (2017) Gastric and intestinal proteases resistance of chicken acidic chitinase nominates chitin‐containing organisms for alternative whole edible diets for poultry. Sci Rep 7, 6662. 28751762 10.1038/s41598-017-07146-3 PMC5532213

[40] Tabata E , Kashimura A , Wakita S , Ohno M , Sakaguchi M , Sugahara Y , Imamura Y , Seki S , Ueda H , Matoska V *et al*. (2017) Protease resistance of porcine acidic mammalian chitinase under gastrointestinal conditions implies that chitin‐containing organisms can be sustainable dietary resources. Sci Rep 7, 12963. 29021549 10.1038/s41598-017-13526-6 PMC5636921

[41] Bucking C and Wood CM (2006) Water dynamics in the digestive tract of the freshwater rainbow trout during the processing of a single meal. J Exp Biol 209, 1883–1893. 16651554 10.1242/jeb.02205

[42] Usher ML , Talbot C and Eddy FB (1990) Effects of transfer to seawater on digestion and gut function in Atlantic salmon smolts (*Salmo salar* L.). Aquaculture 90, 85–96.

[43] Kirsch R and Meister MF (1982) Progressive processing of ingested water in the gut of sea‐water teleosts. J Exp Biol 98, 67–81. 7108442 10.1242/jeb.98.1.67

[44] Huo Y‐Y , Rong Z , Jian S‐L , Xu C‐D , Li J and Xu X‐W (2017) A novel halotolerant thermoalkaliphilic esterase from marine bacterium *Erythrobacter seohaensis* SW‐135. Front Microbiol 8, 2315. 29213264 10.3389/fmicb.2017.02315 PMC5702849

[45] Wang M , Ai L , Zhang M , Wang F and Wang C (2020) Characterization of a novel halotolerant esterase from *Chromohalobacter canadensis* isolated from salt well mine. 3 Biotech 10, 430. 10.1007/s13205-020-02420-0 PMC7490289 32983823

[46] Guo G , Fang T , Wang C , Huang Y , Tian F , Cui Q and Wang H (2015) Isolation and characterization of two novel halotolerant Catechol 2, 3‐dioxygenases from a halophilic bacterial consortium. Sci Rep 5, 17603. 26621792 10.1038/srep17603 PMC4664950

[47] Vaaje‐Kolstad G , Horn SJ , Sørlie M and Eijsink VGH (2013) The chitinolytic machinery of *Serratia marcescens* – a model system for enzymatic degradation of recalcitrant polysaccharides. FEBS J 280, 3028–3049. 23398882 10.1111/febs.12181

[48] Monreal J and Reese ET (1969) The chitinase of *Serratia marcescens* . Can J Microbiol 15, 689–696. 4894282 10.1139/m69-122

[49] Várnai A , Siika‐Aho M and Viikari L (2013) Carbohydrate‐binding modules (CBMs) revisited: reduced amount of water counterbalances the need for CBMs. Biotechnol Biofuels 6, 30. 23442543 10.1186/1754-6834-6-30 PMC3599012

[50] Madland E , Forsberg Z , Wang Y , Lindorff‐Larsen K , Niebisch A , Modregger J , Eijsink VGH , Aachmann FL and Courtade G (2021) Structural and functional variation of chitin‐binding domains of a lytic polysaccharide monooxygenase from *Cellvibrio japonicus* . J Biol Chem 297, 101084. 34411561 10.1016/j.jbc.2021.101084 PMC8449059

[51] Forsberg Z and Courtade G (2023) On the impact of carbohydrate‐binding modules (CBMs) in lytic polysaccharide monooxygenases (LPMOs). Essays Biochem 67, 561–574. 36504118 10.1042/EBC20220162 PMC10154629

[52] Belghit I , Liland NS , Gjesdal P , Biancarosa I , Menchetti E , Li Y , Waagbø R , Krogdahl Å and Lock E‐J (2019) Black soldier fly larvae meal can replace fish meal in diets of sea‐water phase Atlantic salmon (*Salmo salar*). Aquaculture 503, 609–619.

[53] Li Y , Kortner TM , Chikwati EM , Belghit I , Lock E‐J and Krogdahl Å (2020) Total replacement of fish meal with black soldier fly (*Hermetia illucens*) larvae meal does not compromise the gut health of Atlantic salmon (*Salmo salar*). Aquaculture 520, 734967.

[54] Ringø E , Zhou Z , Olsen RE and Song SK (2012) Use of chitin and krill in aquaculture – the effect on gut microbiota and the immune system: a review. Aquacult Nutr 18, 117–131.

[55] Ngo D‐H and Kim S‐K (2014) Antioxidant effects of chitin, chitosan, and their derivatives. In Marine Carbohydrates: Fundamentals and Applications, Part B ( Kim SK , ed.), pp. 15–31. Elsevier, Oxford. 10.1016/B978-0-12-800268-1.00002-0 25300540

[56] Steentoft C , Vakhrushev SY , Joshi HJ , Kong Y , Vester‐Christensen MB , Schjoldager KT‐BG , Lavrsen K , Dabelsteen S , Pedersen NB , Marcos‐Silva L *et al*. (2013) Precision mapping of the human O‐GalNAc glycoproteome through SimpleCell technology. EMBO J 32, 1478–1488. 23584533 10.1038/emboj.2013.79 PMC3655468

[57] Mekasha S , Byman IR , Lynch C , Toupalová H , Anděra L , Næs T , Vaaje‐Kolstad G and Eijsink VGH (2017) Development of enzyme cocktails for complete saccharification of chitin using mono‐component enzymes from *Serratia marcescens* . Process Biochem 56, 132–138.

[58] Shevchenko A , Tomas H , Havlis J , Olsen JV and Mann M (2006) In‐gel digestion for mass spectrometric characterization of proteins and proteomes. Nat Protoc 1, 2856–2860. 17406544 10.1038/nprot.2006.468

[59] Tuveng TR , Arntzen MØ , Bengtsson O , Gardner JG , Vaaje‐Kolstad G and Eijsink VGH (2016) Proteomic investigation of the secretome of *Cellvibrio japonicus* during growth on chitin. Proteomics 16, 1904–1914. 27169553 10.1002/pmic.201500419

[60] Cox J and Mann M (2008) MaxQuant enables high peptide identification rates, individualized p.p.b.‐range mass accuracies and proteome‐wide protein quantification. Nat Biotechnol 26, 1367–1372. 19029910 10.1038/nbt.1511

[61] Cox J , Hein MY , Luber CA , Paron I , Nagaraj N and Mann M (2014) Accurate proteome‐wide label‐free quantification by delayed normalization and maximal peptide ratio extraction, termed MaxLFQ. Mol Cell Proteomics 13, 2513–2526. 24942700 10.1074/mcp.M113.031591 PMC4159666

[62] Lien S , Koop BF , Sandve SR , Miller JR , Kent MP , Nome T , Hvidsten TR , Leong JS , Minkley DR , Zimin A *et al*. (2016) The Atlantic salmon genome provides insights into rediploidization. Nature 533, 200–205. 27088604 10.1038/nature17164 PMC8127823

[63] Dobin A , Davis CA , Schlesinger F , Drenkow J , Zaleski C , Jha S , Batut P , Chaisson M and Gingeras TR (2013) STAR: ultrafast universal RNA‐seq aligner. Bioinformatics 29, 15–21. 23104886 10.1093/bioinformatics/bts635 PMC3530905

[64] Liao Y , Smyth GK and Shi W (2014) featureCounts: an efficient general purpose program for assigning sequence reads to genomic features. Bioinformatics 30, 923–930. 24227677 10.1093/bioinformatics/btt656

[65] Sprotocols (2014) A spinnable and automatable Stagetip for high throughput peptide desalting and proteomics. *Zenodo*.

[66] Hamre AG , Strømnes A‐GS , Gustavsen D , Vaaje‐Kolstad G , Eijsink VGH and Sørlie M (2019) Treatment of recalcitrant crystalline polysaccharides with lytic polysaccharide monooxygenase relieves the need for glycoside hydrolase processivity. Carbohydr Res 473, 66–71. 30640029 10.1016/j.carres.2019.01.001

[67] Vizcaíno JA , Côté RG , Csordas A , Dianes JA , Fabregat A , Foster JM , Griss J , Alpi E , Birim M , Contell J *et al*. (2013) The PRoteomics IDEntifications (PRIDE) database and associated tools: status in 2013. Nucleic Acids Res 41, D1063–D1069. 23203882 10.1093/nar/gks1262 PMC3531176

